# Increased resilience and a regime shift reversal through repeat mass coral bleaching

**DOI:** 10.1111/ele.14454

**Published:** 2024-12-31

**Authors:** Nicholas A. J. Graham, Shaun K. Wilson, Cassandra E. Benkwitt, Rodney Bonne, Rodney Govinden, James P. W. Robinson

**Affiliations:** ^1^ Lancaster Environment Centre Lancaster University Lancaster UK; ^2^ Australian Institute of Marine Science Crawley Western Australia Australia; ^3^ Oceans Institute, University of Western Australia Crawley Western Australia Australia; ^4^ Seychelles Parks and Gardens Authority Victoria Mahe Seychelles; ^5^ Seychelles Fishing Authority Victoria Mahe Seychelles

**Keywords:** beta diversity, coral reef ecology, ecosystem function, herbivory, Indian Ocean, marine heatwave, recovery, reef fish, resistance

## Abstract

Ecosystems are substantially changing in response to ongoing climate change. For example, coral reefs have declined in coral dominance, with some reefs undergoing regime shifts to non‐coral states. However, reef responses may vary through multiple heat stress events, with the rarity of long‐term ecological datasets rendering such understanding uncertain. Assessing coral reefs across the inner Seychelles islands using a 28‐year dataset, we document faster coral recovery from the 2016 than the 1998 marine heatwave event. Further, compositions of benthic and fish communities were more resistant to change following the more recent heat stress, having stabilized in a persistent altered state, with greater herbivory, following the 1998 climate disturbance. Counter to predictions, a macroalgal‐dominated reef that had regime‐shifted following the 1998 disturbance is transitioning to a coral‐dominated state following the 2016 heat stress. Collectively, these patterns indicate that reef systems may be more resilient to repeat heatwave events than anticipated.

## Graphical abstract

Assessing coral reefs across the inner Seychelles islands, using a 28‐year dataset, we document faster coral recovery from the 2016 than the 1998 marine heatwave events. Further, a reef that had regime‐shifted to macroalgal dominance following the 1998 disturbance is transitioning to a coral‐dominated state following the 2016 heat stress. Collectively, these patterns indicate that reef systems may be more resilient to repeat heatwave events than anticipated.

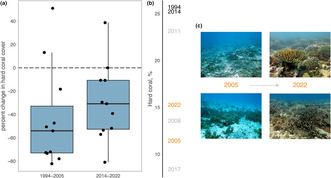

## PEER REVIEW

The peer review history for this article is available at https://www.webofscience.com/api/gateway/wos/peer‐review/10.1111/ele.14454.

## INTRODUCTION

Climate change, rising demands for food and resources, pollution and globalization are driving widespread degradation of terrestrial and aquatic ecosystems (Díaz et al., 2019). Changes include declines in dominant habitat forming organisms, such as trees or corals (Barlow et al., 2018), substantial community turnover (Dornelas et al., 2023) and in some cases shifts to fundamentally different community configurations (Folke et al., 2004). Climate shocks, such as heatwaves, can precipitate abrupt shifts in biodiversity and ecosystem functioning (Smale et al., 2019), and are becoming increasingly pervasive (Perkins‐Kirkpatrick & Lewis, 2020), but there is limited understanding of how ecosystems respond to repeated events.

Coral reefs provide a powerful opportunity to explore this issue. Climate warming, overfishing and poor water quality have caused declines in coral cover, structural complexity and biodiversity (Alvarez‐Filip et al., 2009; Eddy et al., 2021; Pratchett et al., 2008), and marine heatwaves that cause mass coral mortality are increasing in frequency and severity (Hughes et al., 2018; Oliver et al., 2018). These repeated and severe climate shocks are expected to ratchet down coral cover and increase cover of alternate benthic taxa such as algae (Graham et al., 2015; Tebbett et al., 2023). Conversely, changes to thermal tolerance or ecological processes may alter responses to repeat heat stress events such that some reefs are more resilient than expectations (Bay et al., 2017; Guest et al., 2012; Lachs et al., 2023). However, there are few detailed long‐term ecological studies through successive climate disturbances that provide empirical evidence of how coral reef ecosystems respond to repeat severe climate shocks.

Major bleaching events also result in a reshuffling of coral and fish community composition, even if reefs recover to coral‐dominated states (van Woesik et al., 2011; Watt‐Pringle et al., 2022), with altered compositions typically persisting through time (Robinson, Wilson, Jennings, & Graham, 2019). For corals, the frequency of coral bleaching events can determine community composition and vulnerability to repeat heat stress based on differential vulnerability among taxa (Hughes et al., 2021). One commonly reported shift for fishes has been an increase in herbivorous populations, and associated increases in herbivory (Adam et al., 2011; Graham et al., 2020; Taylor et al., 2020), which may influence recovery of corals (Gilmour et al., 2013). However, the extent to which repeat severe climate disturbances reset successional trends in benthic and fish communities, or result in further major reshuffling of community compositions, is poorly known.

Severely disturbed reefs may also undergo regime shifts to non‐calcifying ecosystem states, such as those characterized by high macroalgae, turf algae or sponge cover (Hughes et al., 2017; Norström et al., 2009). Regime shifts are particularly problematic, as they result in major changes to ecosystem functions and services (Folke et al., 2004). Ecological feedbacks maintain systems in regime‐shifted configurations (Nyström et al., 2012), for example macroalgae preventing coral settlement (Chong‐Seng et al., 2014), and as such reversals are unlikely without major changes in the drivers of ecosystem decline. However, theory suggests that shocks to an ecosystem could trigger a regime shift reversal by causing a decline in the organism dominating ecosystem composition (e.g. algae), in a similar way to how shocks trigger regime shifts to less desirable states (Graham et al., 2013).

**FIGURE 1:**
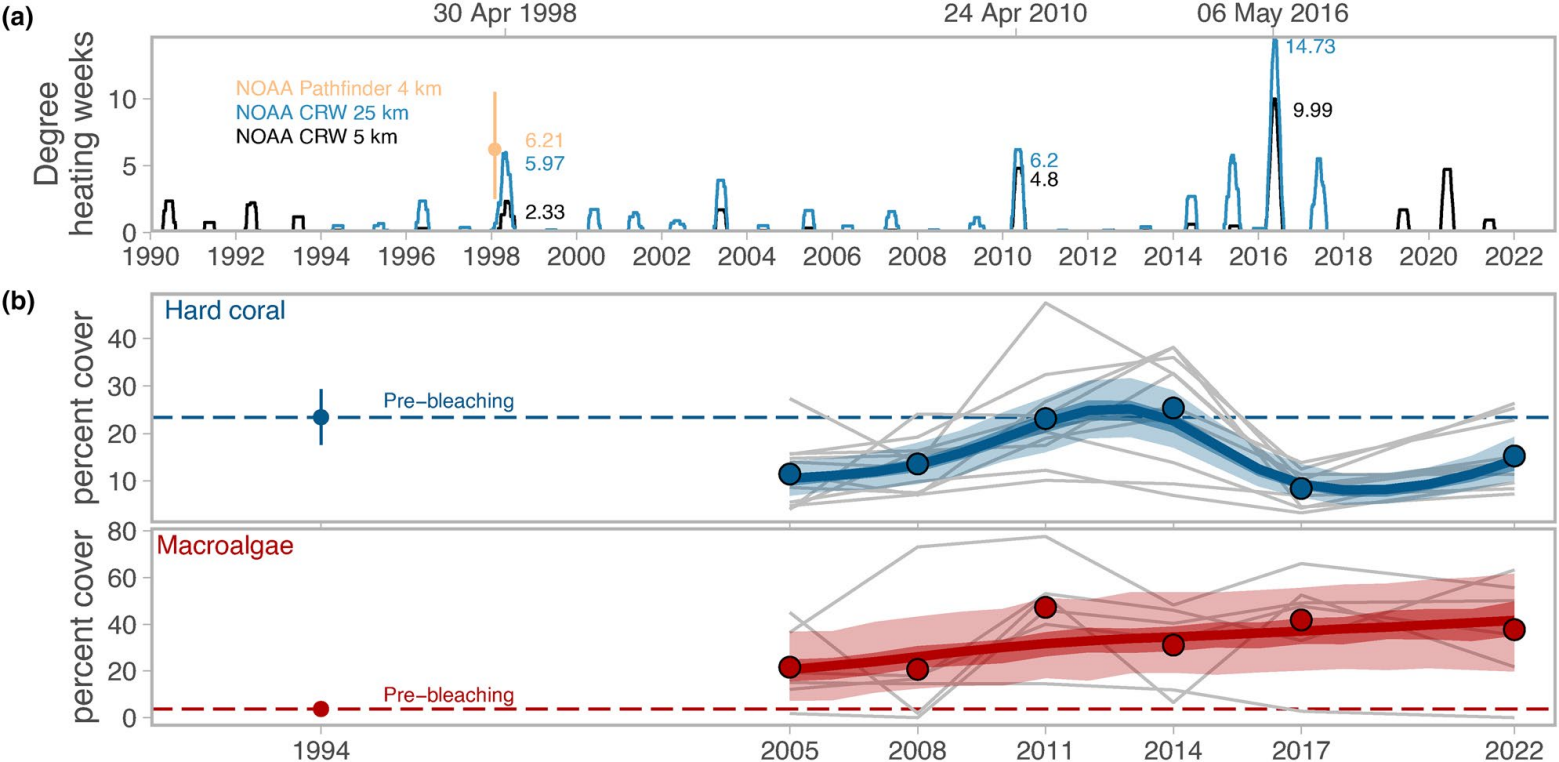
(a) Degree heating weeks 1990–2022 for the inner Seychelles. (b) hard coral cover for 11 reefs that recovered following the 1998 coral bleaching event, and macroalgal cover for six reefs that regime‐shifted to algal dominance following the 1998 coral bleaching event. Coral cover across 22 sites in the inner Seychelles in 2000, shortly after the 1998 coral bleaching event, was estimated at ca. 3% (Engelhardt, 2004). Lines are posterior median predictions for each reef regime (blue = recovering, red = shifted, with 95% and 50% certainty intervals), points show average site‐level values, and grey thin lines are site‐level benthic trajectories. Dashed line is average pre‐bleaching cover, indicated by point (±2. SEM) at 1994.

Here, we use a unique 28‐year case study that tracks benthic and fish community composition on 17 reefs spanning the inner Seychelles islands. During this time, the coral reef system suffered two major marine heatwaves. The 1998 marine heatwave caused an estimated 90% loss of live coral (Goreau et al., 2000), reducing coral cover to an average of ca. 3% (Engelhardt, 2004). Subsequently, about half the reefs regime‐shifted to macroalgal‐dominated systems, while half recovered live coral cover (Graham et al., 2015). Fish community composition changed substantially and stabilized, with an increased dominance of herbivores (Graham et al., 2020; Robinson, Wilson, Jennings, & Graham, 2019). We predicted that subsequent severe bleaching events would cause further degradation, with more reefs undergoing regime shifts from coral to macroalgal dominance (Arif et al., 2022; Januchowski‐Hartley et al., 2017). In 2016, another major marine heatwave caused severe coral bleaching and mortality in the inner Seychelles (Wilson et al., 2019), with the degree heating week stress in excess of 1998 (Figure 1a). Comparing benthic communities before and 6–7 years after these two severe climate disturbance events, we assess how coral reefs responded to repeat mass coral bleaching, consider evidence of further regime shifts, or regime shift reversals, and determine how fish communities have changed in response to these repeat climate disturbances.

## MATERIALS AND METHODS

### Study location and data collection

We use a 28‐year monitoring dataset to evaluate how coral reefs of the inner Seychelles responded to major marine heatwave events in 1998 and 2016. The monitoring dataset started in 1994, before the 1998 heatwave, and was first repeated in 2005, 7 years after that event (Graham et al., 2006). From 2005, sites were monitored every 3 years until 2017 (i.e. 2005, 2008, 2011, 2014 and 2017), with the most recent survey in 2022 (a 5‐year gap in monitoring due to COVID‐19 travel restrictions). This sampling therefore allows meaningful ecological change to be detected over decadal timescales. Further, the 2005 and 2022 survey years provide similar time windows after the 1998 and 2016 marine heatwave events, to compare ecosystem responses.

Following the 1998 bleaching event, four complementary metrics were used to identify reefs as either recovering or regime shifted. The 11 recovering reefs had greater post‐disturbance coral than macroalgal cover, coral cover remaining high or was increasing at a rate exceeding macroalgae, and benthic communities were returning towards pre‐disturbance compositions (Graham et al., 2015). Conversely, the six reefs regime shifted reef had greater post‐disturbance macroalgal than coral cover, macroalgal cover remaining high or was increasing at a rate exceeding coral, and community compositions continued to change (Graham et al., 2015). Recovering reefs returned to coral cover levels similar to pre‐1998 by 2011 or 2014, while regime shifted reefs experienced increases in macroalgae and remained in low coral states with carbonate budgets in net erosion (Graham et al., 2015; Januchowski‐Hartley et al., 2017). We use these benthic trajectories (recovering and regime‐shifted) to understand ecological effects of successive heatwaves on Seychelles' coral reefs.

Benthic and fish communities were surveyed at each site (map in Wilson et al., 2012) by three experienced divers, using identical survey techniques, in each year. Surveys were conducted during the same period each year (March–April) to avoid confounding issues such as seasonal fluctuations in macroalgal cover. Eight to 16 replicates of 7 m radius point counts were surveyed along the reef slope, covering up to 0.5 km of reef front and 2500 m^2^ of reef habitat. Within each point count area, the percent cover of live hard coral by growth form, soft coral, macroalgae, sand, rubble and rock/pavement was estimated visually from a planar view. Coral and algal cover was also estimated at the genera level using 10 m line intercept transects within each point count area. Coral and algal cover estimates were consistent between the visual and line intercept methods (Wilson et al., 2007).

Abundance and individual body length (to the nearest cm) of diurnally active non‐cryptic fish (134 species from 16 families) were estimated using underwater visual census (UVC) within each point count area. Larger, mobile species were recorded before smaller, more site‐attached species to minimize double‐counting. Survey time varied according to the abundance and diversity of fish in each replicate. Fish body length estimations were calibrated at the start of each sampling day in all sample years, and found to be consistently within 4% of actual lengths. Individual fish were converted to biomass with published length–weight relationships (Froese & Pauly, 2022; Letourneur et al., 1998), and fish were placed into trophic groups based on feeding preferences (Wilson et al., 2007).

We estimated ecological variables from benthic (percent cover of habitat‐forming taxa) and fish surveys (fish biomass, community composition) for each reef site‐year combination. Values were averaged across eight replicates at each site‐year combination. In 1994, 2005 and 2008, each site had 16 replicates, so we randomly sampled eight replicates, thus giving a comparable sampling effort to later years.

### Strength and timing of marine heatwaves

We used satellite remote sensing datasets to assess heat stress experienced by Seychelles' reefs. Degree heating weeks (DHW,°C‐weeks) capture the magnitude and duration of marine heatwaves, where 4°C‐weeks are likely to lead to significant coral bleaching, and 8°C‐weeks is associated with coral mortality (Eakin et al., 2010). We extracted DHW for each reef, using publicly‐available products derived from historical satellite remote sensing data of daily sea surface temperature (Heron et al., 2016; Liu et al., 2014). The spatial resolution of DHW maps has improved through time, meaning that historical SST datasets vary in spatial resolution and temporal extent, and each DHW product has its own specific biases. For example, downscaled DHW products are known to be less accurate pre‐2003 (Little et al., 2022), while coarse spatial grains likely fail to capture small‐scale oceanographic features (e.g. upwelling) (Heron et al., 2016). To capture potential uncertainty in DHW estimates over decadal time‐scales, we collated DHW estimates from 5‐km and 25‐km globally gridded products (Liu et al., 2014) and from published values for Seychelles' reefs at 50‐km (McClanahan et al., 2007) and 4‐km (Heron et al., 2016). We used this set of estimates to assess heat stress for corals from 1990 through to 2022, capturing the 1998 and 2016 mass bleaching events.

### Benthic trends and responses

We used Bayesian models and multivariate approaches to assess temporal change in cover of hard corals and macroalgae between 1994 and 2022. We modelled site‐level benthic cover values using zero‐inflated beta Bayesian models, with a spline‐based smoother for year and a random‐intercept for site. Models were fitted separately for hard coral or macroalgae, and for recovering or regime‐shifted sites, and we used posterior predictive distributions to assess benthic trends from 2005 to 2022, relative to pre‐bleaching values in 1994. We assessed heatwave effects on hard coral cover by estimating site‐level change in hard coral cover 1994 to 2005 (1998 heatwave) and 2014 to 2022 (2016 heatwave), representing a similar time span following both heatwaves (6–7 years). We compared coral recovery rates after each heatwave by refitting the hard coral model with year as a categorical covariate, allowing a probability estimate that 2022 cover was higher than 2005 or 2008.

Temporal trends in benthic composition were estimated using Euclidean distance of each reef relative to its pre‐bleaching year (1994). Each reef's benthic composition was represented by percent cover of biotic variables, here characterized as five hard coral growth forms (branching, massive, table, encrusting, sub‐massive), soft corals and macroalgae (turf algae was not recorded). We repeated this analysis including both biotic and non‐biotic (sand, rock/pavement, rubble) variables. Cover values were log_10_(x + 1) transformed. We further investigated benthic composition by plotting percent cover of the four dominant coral growth forms (branching, encrusting, massive and tables) through time, and plotting a biplot of encrusting coral cover by massive coral cover on recovering reefs 2005–2022. Finally, we analysed compositional change in coral genera (*n* = 41 recorded) on recovering reefs and macroalgal genera (*n* = 13) on regime‐shifted reefs through the 2016 heatwave event (2014, 2017 and 2022 survey years; genera level information unavailable for 1994 and 2005) using non‐metric multidimensional scaling on untransformed data and a Bray Curtis resemblance matrix.

### Fish community trajectories

We used abundance, biomass and biodiversity metrics to assess temporal change in reef fish communities 1994–2022. We estimated average site‐level biomass and abundance of species across point counts. For each reef site and survey year, we then estimated total community fish biomass (kg/ha), and abundance or biomass of five functional groups that are known to associate strongly with reef benthos (herbivore browser, herbivore scraper [includes scraping and excavating parrotfishes], herbivore grazer, small‐bodied planktivores and corallivores) (Pratchett et al., 2008; Robinson, Wilson, Jennings, & Graham, 2019). We further investigated species level patterns in the herbivore community by identifying the top five species by biomass for each year, separately for recovering and regime‐shifted reefs. We assessed compositional change in fish communities by estimating beta diversity of each reef's fish community relative to its pre‐bleaching baseline (1994). Beta metrics were derived from species‐level pairwise biomass‐based comparisons of the Bray‐Curtis index (Baselga & Orme, 2012).

We used Bayesian linear models to quantify temporal trends in biomass (total and functional group), beta‐diversity values and abundance (corallivores). Models had a spline‐based smooth for year, fitted separately for recovering and shifted sites, and site was a random‐intercept. Biomass and abundance models had lognormal distributions and beta‐diversity models had Beta distributions. All Bayesian models were implemented in brms (Bürkner, 2021) with Stan (Stan Development Team, 2020) and R 4.2.0 (R Core Team, 2023). All models sampled four chains with 4000 iterations (warm‐up of 1000). We used trace plots to assess model convergence, ensuring no divergent transitions and that Rhat was within 0.01 of 1.

## RESULTS

Degree Heating Weeks (DHW) were high in 1998 and 2016, corresponding with field observations of mass coral bleaching and mortality. In 1998, a large‐scale 50 km product estimated 14°C‐weeks for the inner Seychelles (McClanahan et al., 2007), whereas recent downscaled products estimated DHW between 2 and 6°C‐weeks (Figure 1a). Despite uncertainty of hindcasting newer downscaled products, all DHW estimates indicate the 2016 event was at least as severe as 1998, and likely more severe, with the 5 and 25 km products giving DHW values of ca. 10 and ca. 15°C‐weeks respectively, with additional warm spikes in 2015 and 2017 (Figure 1a). The next most significant heatwave in the time series was in 2010 (4.8–6.2°C‐weeks in 5 and 25 km products), but this event caused minimal coral mortality (primarily for low‐abundance table Acropora corals) and no detectable decline in total coral cover (Wilson et al., 2019).

Coral loss through the 1998 event was extreme, with average cover across all reefs at 7.5% ± 1.6 SEM by 2005 (Graham et al., 2006), far below pre‐bleaching levels in 1994 (25% ± 2.6; Figure 1b). On reefs that recovered, coral cover returned to pre‐bleaching levels by 2011 and remained high until 2014 (mean 27% ± 3.4; Figure 1b). However, extensive bleaching and mortality during the 2016 heatwave (Wilson et al., 2019) caused coral cover to decline again by 2017 (mean 6% ± 1.1 across all reefs, and 8% ± 1 for reefs that had recovered). Reefs that had previously recovered were already showing signs of recovery by 2022 (mean 15% ± 2; Figure 1b), and macroalgae remained low on these recovering reefs throughout the time series (mean 0.8%, ± 0.4; Figure S1). In contrast, on reefs that underwent a regime shift, coral cover has remained far below pre‐bleaching levels following the 1998 heatwave (mean 4.6% ± 0.8; Figure S1). These shifted reefs experienced gradual increases in macroalgae, which stabilized as the dominant benthic organism (mean ca. 40%) and was generally unaffected by the 2016 marine heatwave (Figure 1b).

**FIGURE 2:**
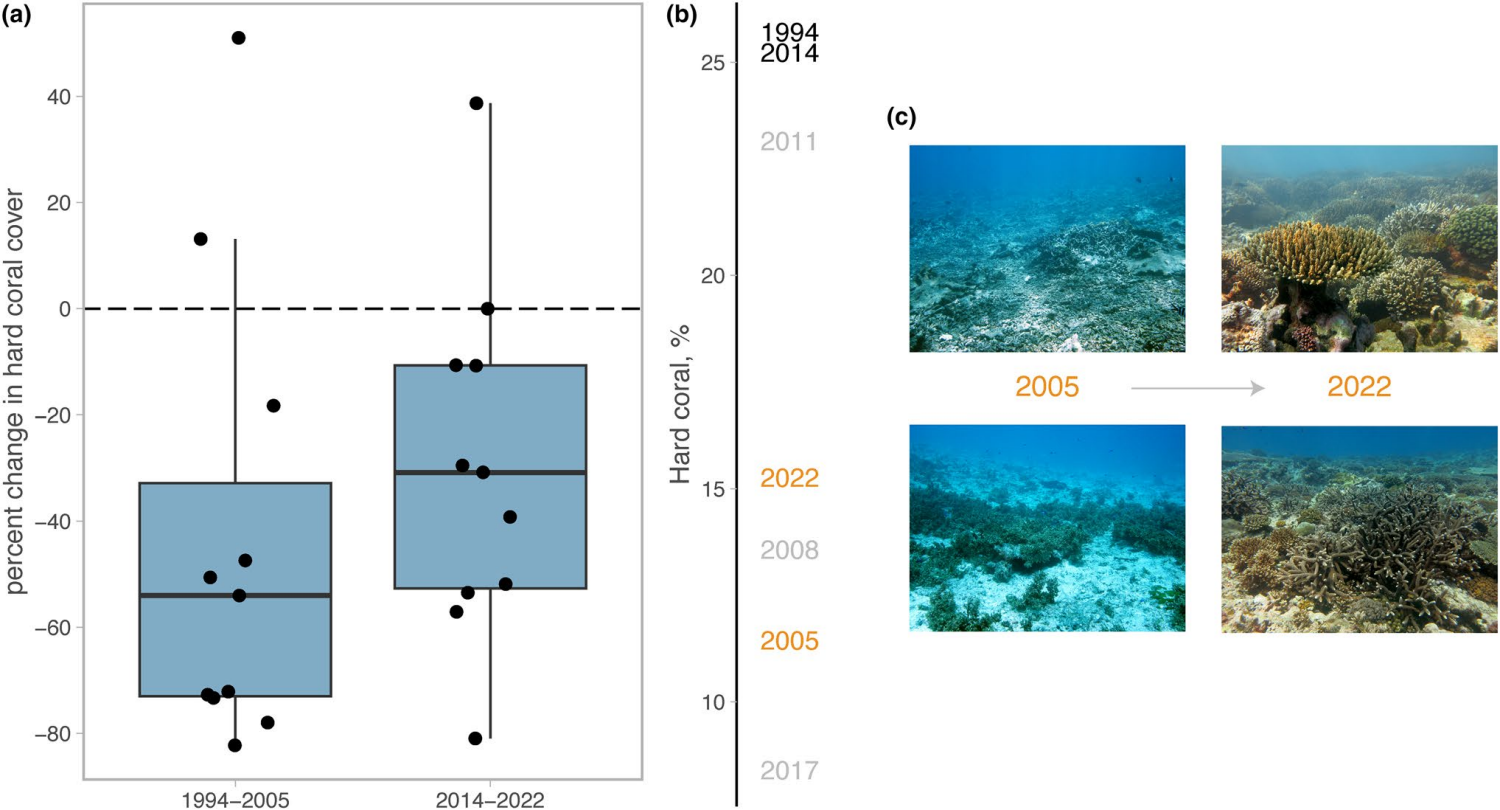
(a) Change in hard coral cover for 11 reefs that recovered following the 1998 coral bleaching event. Change is shown through the 1998 heatwave (1994–2005) and 2016 heatwave (2014–2022). (b) mean coral cover estimate per year for the same 11 reefs, with last year prior to bleaching in black, and the 2 years 6/7 years post bleaching in orange. (c) photos of reef slope habitats on east (upper images) and west (lower images) sides of Cousine Island in 2005 verses 2022.

Surveys conducted in 2005 and 2022 provide insights into medium‐term recovery windows after different severe bleaching events (i.e. 7 years after 1998 and 6 years after 2016, respectively). Relative coral cover was 44% (54% median) lower at recovering reefs in 2005 compared to pre‐1998 values (Figure 2a). Average coral cover in 2005 on reefs that subsequently recovered was 11%. In contrast, for the same reefs, we recorded an average 30% lower coral cover in 2022 compared to 2014 (Figure 2a). Rates and timing of coral cover increase between 2017 and 2022 suggest coral recovery was faster following the second mass bleaching event. Coral cover was ca. 8% in 2017 immediately after the 2016 bleaching event and doubled to ca. 15% by 2022 (6 years after bleaching), which was similar to 2008 levels (ca. 14%, 10 years after the 1998 bleaching) (Figure 2b). Posterior predictions from hard coral models predict that 2022 had an 88% probability of having higher coral cover than 2005, and a 67% probability of higher cover than 2008. This indicates that the reefs were on a steeper recovery trajectory after the second mass bleaching event, with a ca. 4 years faster return of coral cover (relative to 1998–2008 when reefs required 10 years to reach ca. 15% coral cover). For some reefs in the inner Seychelles, this difference in recovery rates was stark, such as reefs on either side of Cousine Island, which had 2.5% average coral cover 7 years after the 1998 bleaching, but had recovered to 39% average coral cover in 2022, 6 years after the 2016 heating event (Figure 2c).

**FIGURE 3:**
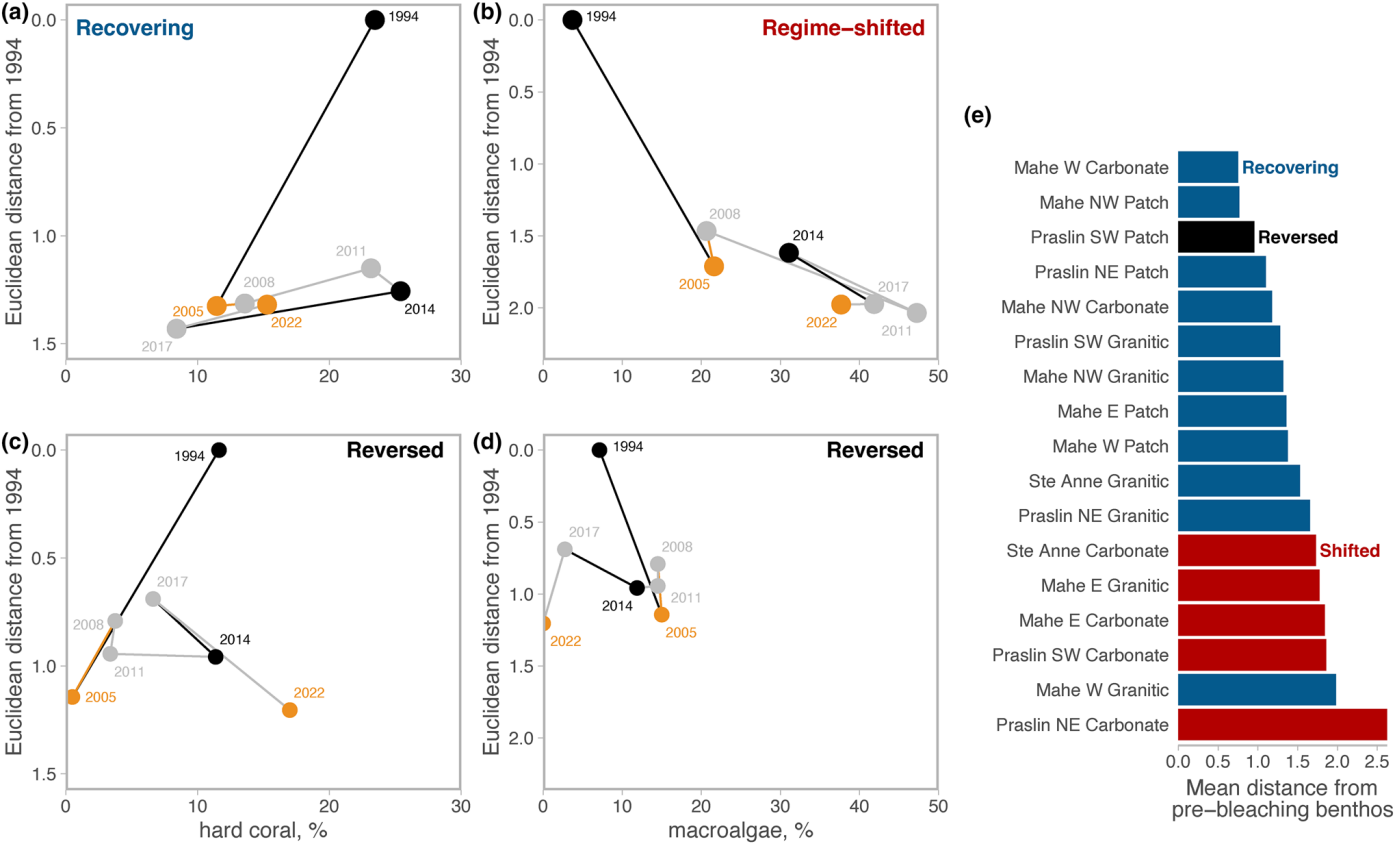
Change in benthic community composition (coral growth forms, soft coral and macroalgal cover) among survey years, with 1994 as the baseline, plotted against percent cover of live hard coral (a and c), and percent cover of macroalgae (b and d), for (a) recovering reefs, (b) regime‐shifted reefs, and (c) and (d) for one reef that has regime‐shift reversed following the 2016 heatwave. e. Average distance from 1994 pre‐bleaching benthic composition for each reef site, with regime‐shifted sites in red and recovering sites in blue.

Recovering reefs underwent substantial changes in benthic community composition following the 1998 heat stress event (Figure 3a). As coral cover returned to pre‐bleaching levels up to 2014, recovering reefs remained dissimilar from pre‐bleaching compositions (Figure 3a). In contrast, the loss in coral cover due to the 2016 bleaching event was accompanied by limited changes in community composition (relative to the preceding pre‐bleaching survey year of 2014), with the recovery trajectory in terms of both coral cover and community composition moving towards the post‐1998 recovery state evident when assessing just biotic or biotic and non‐biotic variables (Figure 3a; Figure S2). Cover of branching, encrusting and massive corals changed after heatwaves and during recovery periods, with branching corals showing pronounced boom‐bust dynamics (Figure S3), while encrusting and massive corals trade‐off in terms of which reefs they occur on (Figure S4). At the genera level, *Acropora* corals (of which the majority are branching in this system) dominated changes in assemblage composition through the 2016 bleaching event, with reefs in 2017 and 2022 declining to lower cover than in 2014 (Figure S5). On some of these reefs, changes in *Acropora* are supplemented by increased cover of massive *Porites* and *Gonipora*, while on other reefs, encrusting *Favites* and *Acanthastrea* increased.

Regime‐shifted reefs also transitioned to new community compositions after the 1998 bleaching event, defined by high macroalgal cover and limited coral recovery (Graham et al., 2015). After the 2016 event, this alternative benthic regime showed little change, with high macroalgal cover and a more altered community composition state than recovering reefs (*y*‐axis range in Figure 3b vs. Figure 3a; Figure S3). The macroalgal assemblage was dominated by *Sargassum* and *Turbinaria*, and experienced limited turnover through the 2016 heatwave (Figure S5).

**FIGURE 4:**
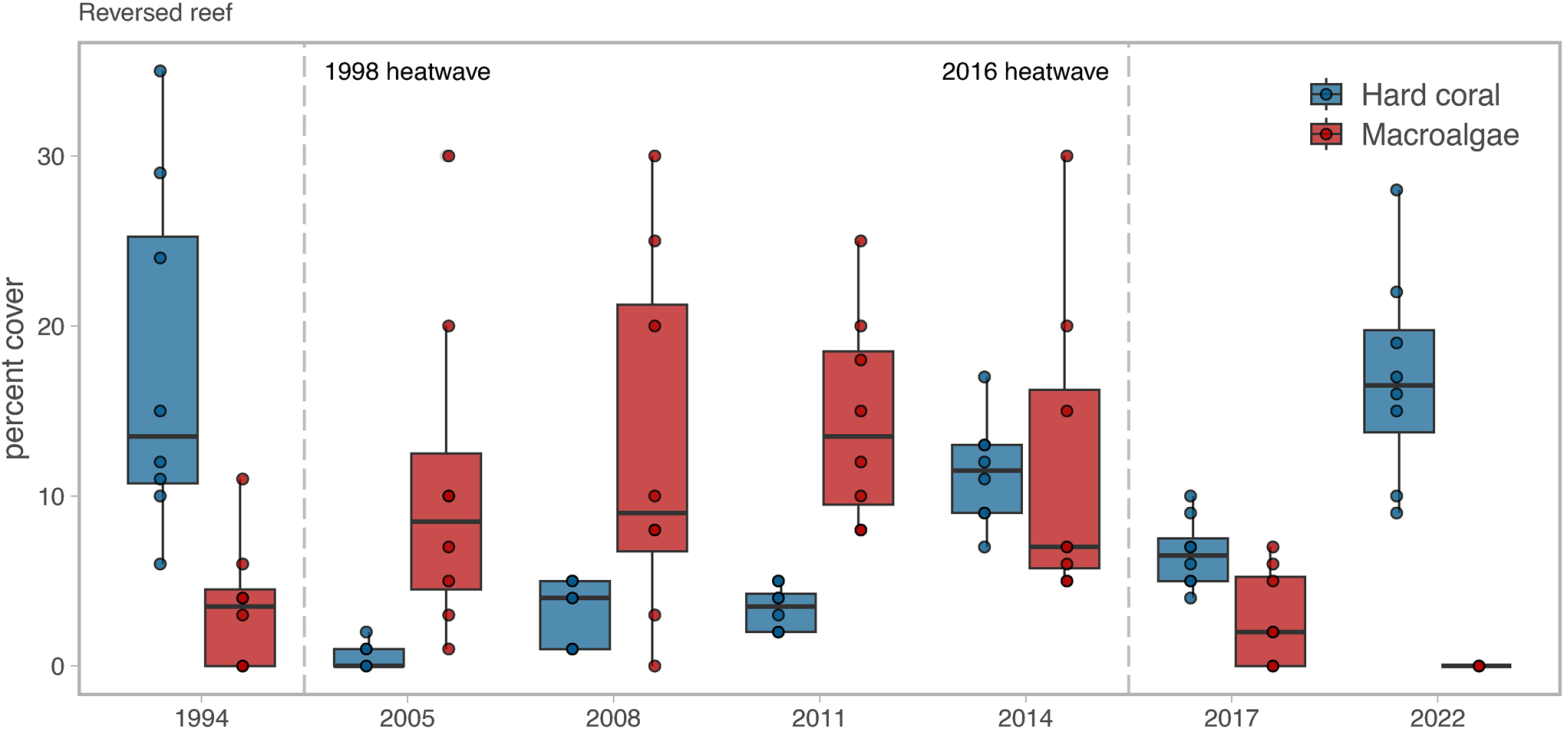
Benthic cover at the reversed reef (Praslin SW Patch) from 1994 to 2022. Boxplots show median and 25th and 75% quantiles of percent cover for hard coral (blue) and macroalgae (red). Boxplot whiskers show the largest values that do not exceed 1.5 * interquantile range, and points show replicate point counts.

The overall stasis in regime‐shifted reef benthos through the second bleaching event belies a rare case of a regime shift reversal in one reef site. This reef was coral dominated in 1994, shifted to macroalgal dominance (mainly *Halimeda*) from 2005 to 2014, but returned to a coral‐dominated regime following the 2016 bleaching event (Figure 4). Four complimentary static and trajectory metrics outlined in Graham et al. (2015) qualify this reef as changing from a regime‐shifted to a recovering reef through the second bleaching event. For example, this reversed reef is on a trajectory towards recovering coral (2022 coral cover at 17%, higher than the average of 15% for all recovering reefs in that year), while macroalgae has disappeared (0% cover; Figures 3c,d, 4). This site also sits firmly at the recovering end of the spectrum of reefs in terms of the mean dissimilarity in community composition in 2022 compared to the 1994 baseline (Figure 3e). Of note, the Mahe W Granite site recovered its coral cover (25% in 2022 compared to 19% in 1994), but has a very dissimilar composition (Figure 3e) due to a large decline in soft coral and increased dominance of encrusting hard corals.

**FIGURE 5:**
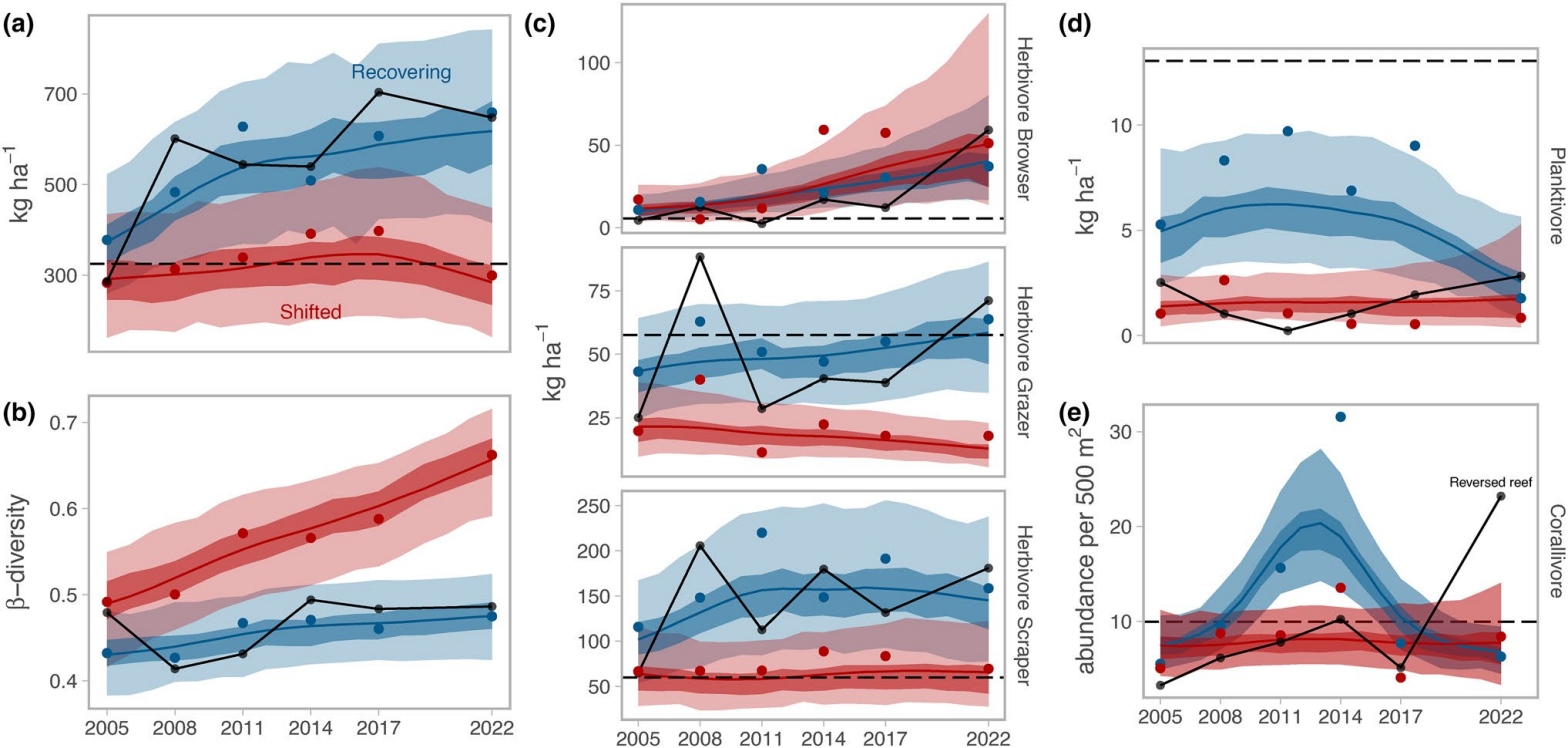
(a) Reef fish biomass, (b) Beta‐diversity, (c) biomass of browsing herbivores, grazing herbivores, and scraping herbivores, (d) biomass of planktivores, and (e) corallivore abundance on recovering verses regime‐shifted reefs 2005–2022, with 1994 baseline value represented as horizontal dashed line and 0 for panel b. Regime shift reversal reef trends shown as black solid line. Lines are median posterior predictions for each reef regime over time (95% and 50% certainty intervals) and points are average site‐level values.

The fish community has responded differently on reefs that recovered versus those that regime‐shifted (Robinson, Wilson, Jennings, & Graham, 2019), and these changes have persisted through the 2016 bleaching event (Figure 5). Standing fish biomass has remained high on recovering reefs, substantially higher than the 1994 baseline, whereas biomass on regime‐shifted reefs remained at 1994 baseline levels (Figure 5a). Interestingly, the beta‐diversity of the fish community largely stabilized following the 1998 bleaching event, particularly for recovering reefs, and showed no marked change through the 2016 event (Figure 5b). Browsing and scraping herbivores drove much of the increase in fish biomass on recovering reefs, resulting in persistently greater herbivory by the 2016 heatwave (Figure 5c). Herbivore grazers also recovered to pre‐1998 baselines on recovering reefs by 2022, but declined through time on regime‐shifted reefs (Figure 5c). Scraping/excavating parrotfish species, such as *Chlorurus sordidus* (Forsskål), *Scarus niger* (Forsskål) and *Scarus rubroviolaceus* (Bleeker), dominated herbivore biomass on both recovering and regime‐shifted reefs, and remained high biomass species particularly on recovering reefs through to 2022. Browsing species, such as *Siganus sutor* (Valenciennes), also became high biomass herbivores on regime‐shifted reefs by 2014 (Table S1). Some groups of fish responded negatively to the bleaching events, with planktivore populations failing to fully recover, and corallivore populations showing boom‐bust dynamics (Figure 5d,e). Interestingly the site that underwent a regime shift reversal followed recovered reef values for standing fish biomass, beta‐diversity, and herbivore biomass (Figure 5a–c). Planktivore and corallivore populations followed regime‐shifted reef trajectories until 2017, after which corallivore populations recovered rapidly to 2022 (Figure 5d,e).

## DISCUSSION

Predictions suggest that many ecosystems will continue to degrade through repeat climate disturbance events (IPCC, 2018). Here, using a long‐term ecological dataset, we assessed coral reef benthic and fish responses to two severe marine heatwaves. We found that all reefs that recovered from the first climate disturbance event are on a recovery trajectory from severe coral mortality again, and that the rate of recovery is substantially faster following the second bleaching event. Both benthic and fish community compositions were more stable through the second severe heat stress event, despite substantial coral cover change. Collectively, these attributes suggest the system was more resilient to the repeat severe climate disturbance, showing both greater resistance to community change and faster recovery of the dominant benthic organism. We also observed a regime shift reversal, whereby one reef that persisted in a macroalgal‐dominated state for 16 years following the 1998 climate disturbance, was recovering to a coral‐dominated regime 6 years after the 2016 marine heatwave. Such regime shift reversals are rarely observed, and this is another indicator that the ecosystem has increased resilience through time.

Persistence of habitat‐forming taxa and recovery rate from disturbance has been of considerable interest to ecologists for decades (Jones & Schmitz, 2009), and is of increasing relevance as climate‐driven disturbances become more frequent and intense (Perkins‐Kirkpatrick & Lewis, 2020). While meta‐analyses have demonstrated that coral recovery rates vary with oceanographic and biological processes (e.g. disturbance type, ocean basin, connectivity) (Graham et al., 2011), few studies have assessed recovery rate of individual reefs to recurring severe disturbances. Here, we have shown how early coral recovery rates in the inner Seychelles were faster in response to the most recent mass coral bleaching event. In contrast, a long‐term study in the Lakshadweep archipelago documented increased resistance of corals to heat stress through the 1998, 2010 and 2016 heatwave events, but reduced recovery rate following the 2010 event compared to 1998 (Yadav et al., 2018). This change in recovery rate was most likely due to a change in coral composition with reduced abundance of Acropora corals and increased dominance of slow‐growing Porites (Yadav et al., 2018). In Seychelles, fast‐growing Acropora and Pocillopora corals have been driving temporal dynamics in coral cover (Wilson et al., 2019), suggesting processes other than large compositional shifts are likely responsible for the increase in recovery rate. While the recovery window of 6–7 years after bleaching precluded us from assessing the stabilization of coral cover, this time window aligns with the predicted frequency of repeat coral bleaching events (Hughes et al., 2018) and thus provides insights into likely benthic trajectories for climate‐impacted coral reefs.

There are likely several reasons for the faster coral recovery rates we documented here. Genomic models (Bay et al., 2017), laboratory experiments (Quigley et al., 2020) and field tests (Palumbi et al., 2014) all suggest coral acclimation and adaptation to repeat heat stress is likely. While further research would be needed to uncover these mechanisms on Seychelles reefs, changes in thermal tolerance appear to be grounded at large spatial scales, with an increasing number of studies finding reduced coral mortality from repeat (and often more severe) heat stress (Fox et al., 2021; González‐Barrios et al., 2023; Hughes et al., 2021; Yadav et al., 2018). While some of this reduction in mortality is likely due to vulnerable coral taxa already being lower in abundance, some is also reflected in the same taxa coping better with the second heat stress (McClanahan, 2017). Survival and proliferation of thermally tolerant corals following heat stress should result in a greater broodstock of adult corals remaining in the wider seascape. In Seychelles, coral cover on recovering reefs was 8% in 2017, whereas immediately after 1998 coral cover in the inner Seychelles was estimated at ca. 3% (Engelhardt, 2004). It therefore seems highly likely that greater adult broodstock survived following the 2016 heatwave, providing reefs with a greater supply of larval corals. This is particularly important for inner Seychelles reefs that are relatively isolated from other potential larval sources (Burt et al., 2024). Another key driver of coral reef recovery potential is herbivory, which controls algal overgrowth and provides grazed substrate suitable for coral settlement (Gilmour et al., 2013; Mumby et al., 2007). On Seychelles' reefs, herbivore populations have increased following the 1998 coral bleaching event, with 2016 biomass estimates of browsing and scraping fishes ca. 184 kg/ha higher than pre‐disturbance levels. This is despite a small increase in fishing pressure during the same period (Robinson, Wilson, Robinson, et al., 2019). Greater herbivory has likely facilitated faster colonization and establishment of newly settling corals, and herbivore biomass is a key determinant of whether these reefs bounce back from severe disturbance (Graham et al., 2015).

Temporal change in community composition has emerged as a key pattern of change in local‐scale biodiversity research (Dornelas et al., 2023), yet understanding of community turnover through repeat climate shocks is limited. The community compositions of benthic and fish assemblages were both more resistant to change following the 2016 heatwave event, suggesting that these communities are more resistant to future shocks. For the benthos, increases in encrusting coral cover after 2005 supports findings that these growth forms do well on climate disturbed reefs (Morgan & Kench, 2017), although they could also have been poorly sampled at low abundance in earlier years. While encrusting corals increased on some reefs, massive corals did on others. Trade‐offs between growth forms were likely related to suitability of benthic substrate type. Major fluctuations in coral cover since 1998 have been driven largely by branching Acropora corals, and the reefs are also characterized by greater rubble and algae coverage (Wilson et al., 2019). While branching Acropora corals drive large changes in coral cover, their initial recovery rate following mortality can be slow, reflecting declines in post‐disturbance juvenile coral densities (Dajka et al., 2019), reliant on larval supply and successful settlement (Chong‐Seng et al., 2014).

For fishes, herbivores have been the big winners, responding to abundant algal resources (Robinson, Wilson, Jennings, & Graham, 2019), with increased productivity translating to a stabilization at higher biomass than pre‐1998 (Hamilton et al. 2022). Browsing herbivores that feed on macroalgae increased in biomass, suggesting their populations are changing in response to newly abundant macroalgae, but are not at sufficient population sizes to reduce algal dominance on many reefs, likely in part due to browsers being a major target of trap fisheries in Seychelles (Robinson, Wilson, Robinson, et al., 2019). Scraping parrotfish, that clear away the epilithic algal matrix facilitating coral recruitment and recovery dynamics (Mumby et al., 2007), increased on recovering reefs. We hypothesize that these scrapers were at sufficient biomass to promote repeat recovery at the recovering sites and enable recovery following the 2016 marine heatwave at the regime shift reversal site. Herbivorous grazers recovered to pre‐bleaching biomass on recovering reefs and the regime shift reversal reef (>57 kg/ha) by 2022, but have steadily declined on regime‐shifted reefs (< 25 kg/ha), further suggesting herbivory may be supporting coral populations on the recovering reefs. These herbivore‐dominated reef fish assemblages appear quite stable to future disturbances, with implications for fisheries and protected area management (Graham et al., 2020; Robinson, Wilson, Robinson, et al., 2019).

Based on how reefs responded to the 1998 disturbance, and the predictors of recovery trajectories, we anticipated regime‐shifted reefs would remain dominated by macroalgae, and that some coral‐dominated reefs would also shift to algal‐dominance following the 2016 disturbance (Arif et al., 2022; Januchowski‐Hartley et al., 2017). Instead, all reefs that recovered following 1998 have remained on a coral recovery trajectory, while one macroalgal regime‐shifted reef is now recovering towards hard‐coral dominance. The complete disappearance of macroalgae from this site is in stark contrast to the other five regime shifted sites that remained dominated by macroalgae (average cover in 2022 = 45%). Longer time‐series will be needed to confirm full coral recovery, but collectively these data suggest a regime shift reversal. Such regime shift reversals are rare in the literature, and thus likely rare in the natural world. Indeed, most studies of terrestrial and aquatic ecosystems have documented regime shifts away from historically dominant biological assemblages (Folke et al., 2004), while examples of regime shift reversals are typically from experimental systems, such as small freshwater lakes where nutrient inputs and fish densities have been manipulated (Scheffer et al., 2001). Some coral reef sites have shown potential for regime shift reversals in the Caribbean, often following a recovery of key processes such as herbivory by sea urchins or survival of key structural features (Carpenter & Edmunds, 2006; Idjadi et al., 2006), but the stability of these states are uncertain (Quinn & Kojis, 2008).

Theory and experimental evidence predicts that regime shifts are persistent because of ecological feedbacks, meaning the drivers of shifts need to be reduced beyond the point of the initial change; a phenomenon known as hysteresis (Scheffer & Carpenter, 2003). Environmental shocks can disrupt resilience, including that of undesirable states (Graham et al., 2013). Indeed, aquatic plants, such as kelp and seagrass, are highly vulnerable to die offs during marine heatwaves (Smale et al., 2019; Strydom et al., 2020; Wernberg et al., 2016). Tropical macroalgae such as Sargassum and Halimeda, which dominate regime‐sifted reefs in Seychelles, can also exhibit reduced growth and mass mortality to heat stress events (Anton et al., 2020; Graba‐Landry et al., 2020; Wei et al., 2020). It is therefore possible that benthic space was made available through macroalgal die‐off in the extreme marine heatwave conditions of 2016. The regime shift reversal reef was already showing signs of recovery potential, with coral cover increasing in 2014 despite macroalgae presence (Figure 4), and some of the core fish variables following recovering reef trajectories (Figure 5). These factors, along with higher herbivory in the system and a faster rate of coral recovery in general post‐2016, may have facilitated the regime shift reversal.

This is a rare example of a vulnerable ecosystem increasing in resilience to repeat climate shocks, despite widely held expectations of ecosystem collapse. Predictions about increasing thermal tolerance may be reflected in how the system is responding to repeat heat stress events, likely coupled with the greater herbivory observed which will provide suitable conditions for coral recovery. Although severe and frequent coral bleaching events will likely degrade many reefs into low coral cover states if climate warming increases unabated, our results give some reason to be optimistic that meeting ambitious climate targets could result in some coral reefs persisting.

## AUTHOR CONTRIBUTIONS

NAJG and SKW designed the ecological surveys and collected the data with CEB, RG and RB. JPWR and NAJG analysed the data. NAJG led the initial draft of the manuscript. All authors edited subsequent drafts.

## Supporting information


Figure S1.

Figure S2.

Figure S3.

Figure S4.

Figure S5.

Table S1.

## Data Availability

The data and code that support the findings of this study are openly available in zenodo at https://zenodo.org/records/10160996, reference number [10160996].
