# Supplementary figures and images for: Increased resilience and a regime shift reversal through repeat mass coral bleaching
Source: Ecol Lett. 2024 Dec 31;27(12):e14454. doi: 10.1111/ele.14454 (PMC11686943; doi:10.1111/ele.14454)

**a**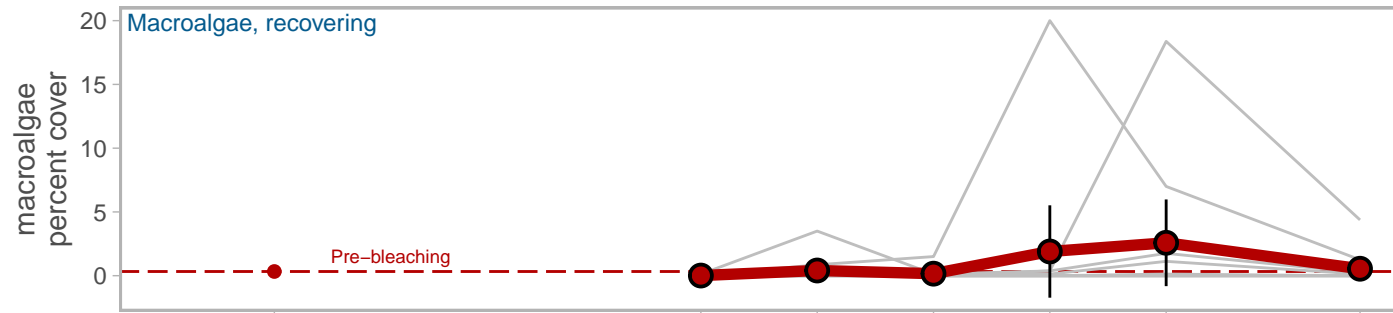**b**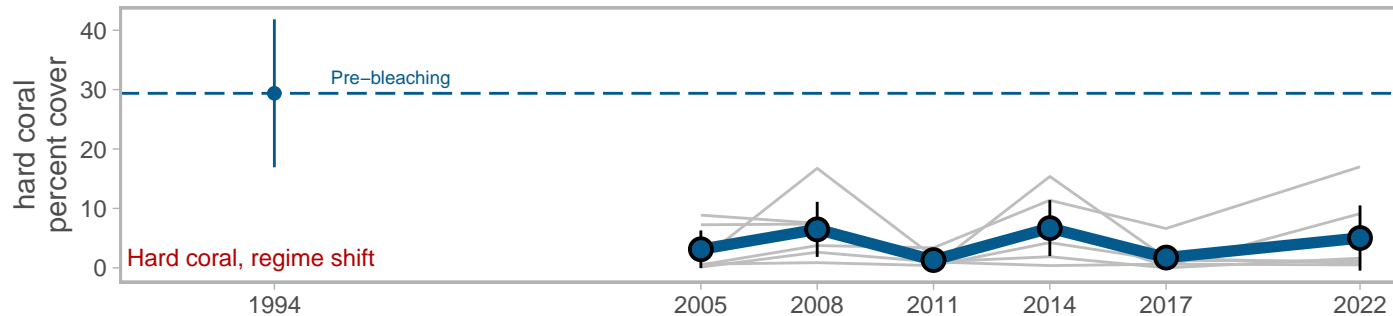

Supplement: Supplementary file 1 — Figure S1. [file ELE-27-0-s005.pdf]

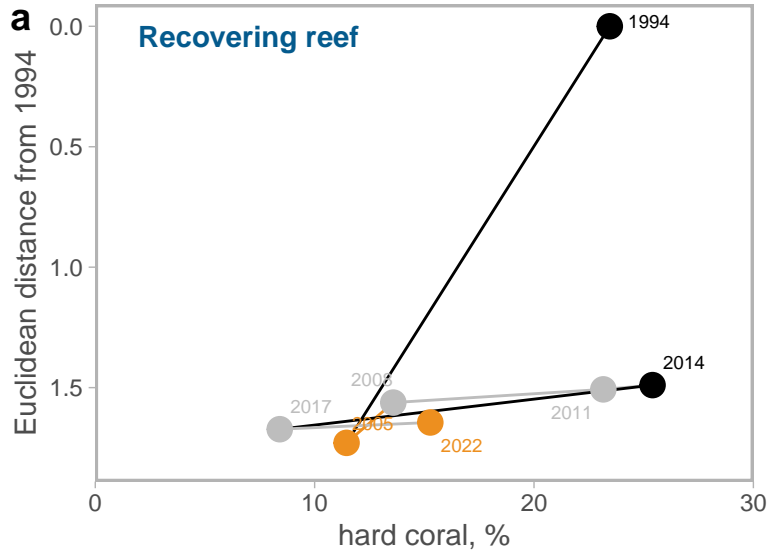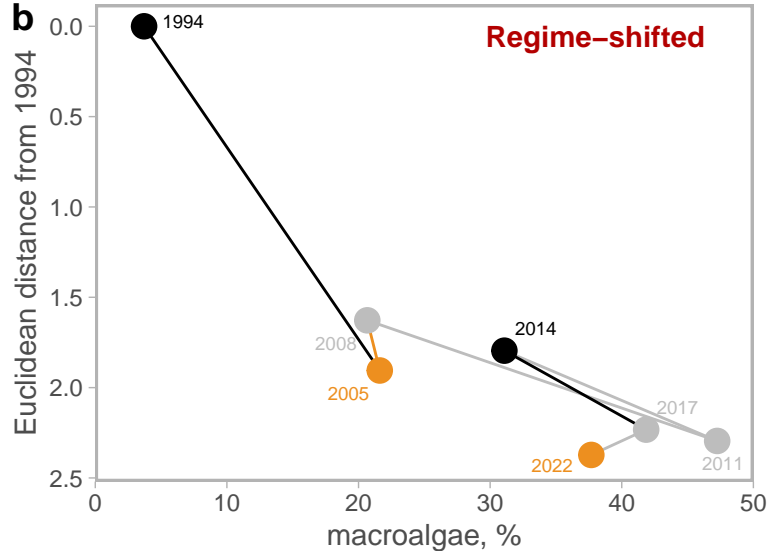

Supplement: Supplementary file 2 — Figure S2. [file ELE-27-0-s004.pdf]

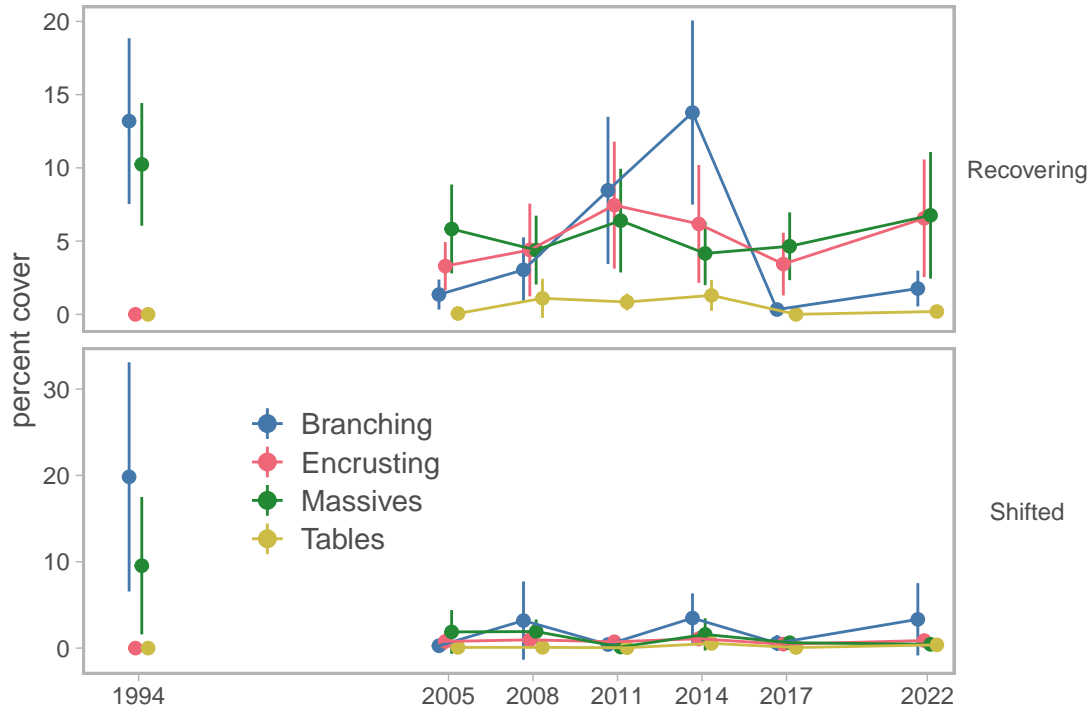

Supplement: Supplementary file 3 — Figure S3. [file ELE-27-0-s001.pdf]

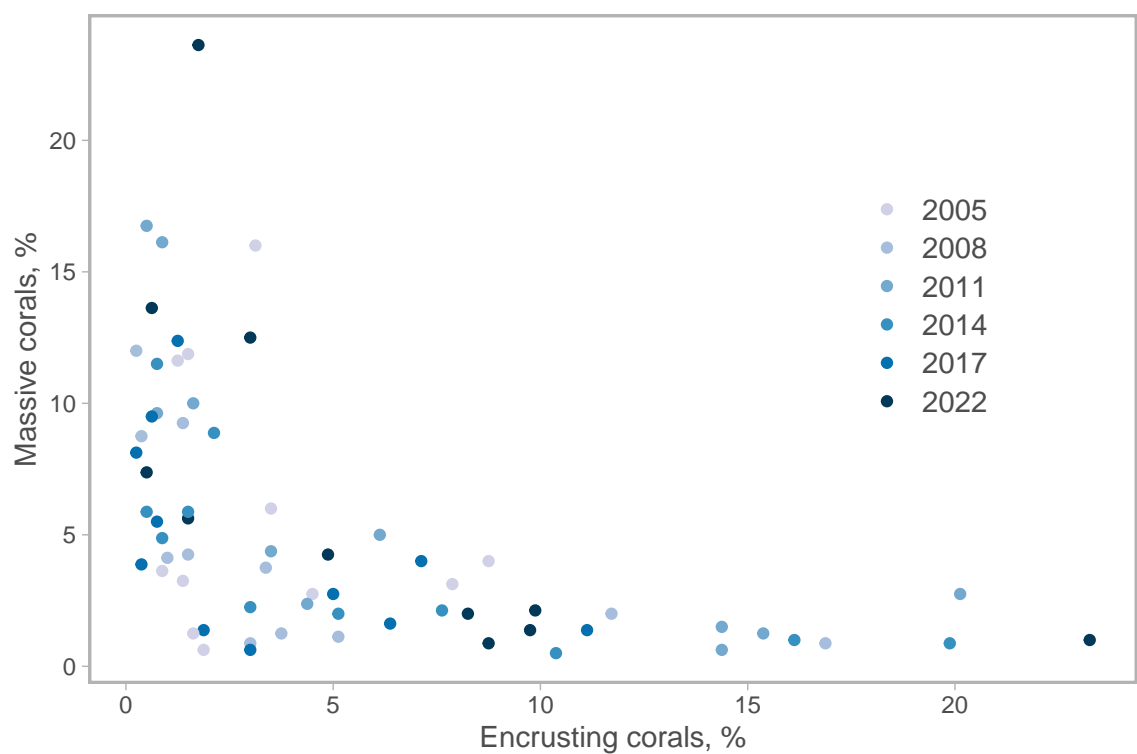

Supplement: Supplementary file 4 — Figure S4. [file ELE-27-0-s003.pdf]

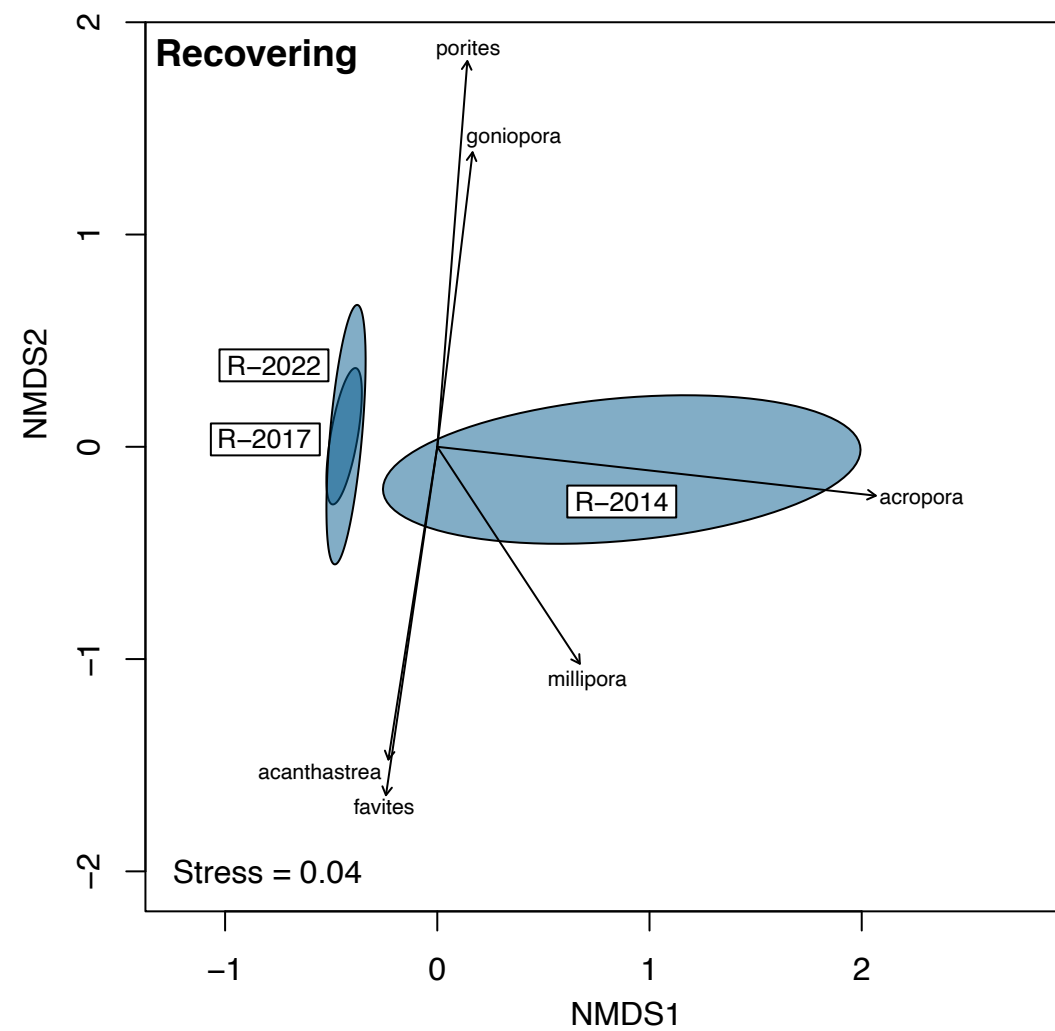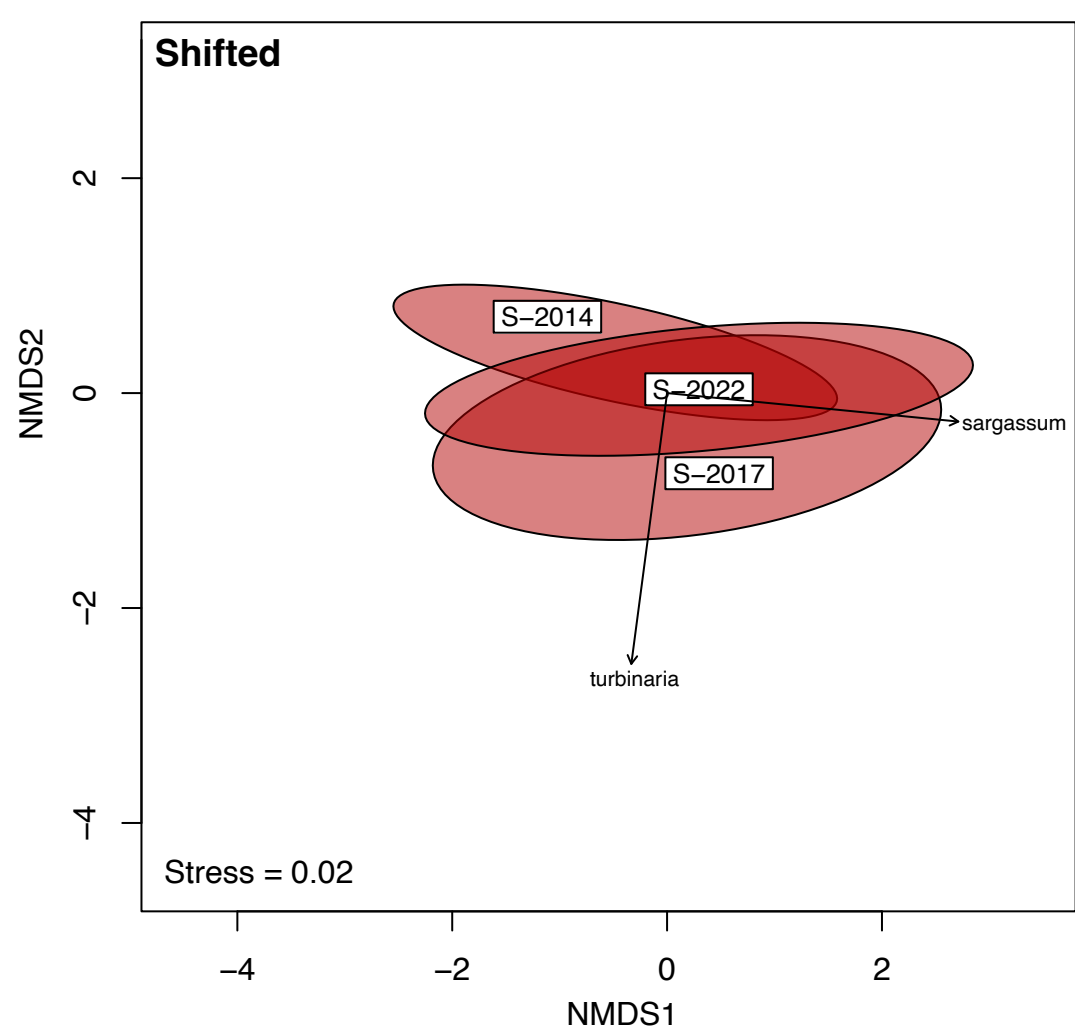

Supplement: Supplementary file 5 — Figure S5. [file ELE-27-0-s006.pdf]
