# Supplementary material for: Increased resilience and a regime shift reversal through repeat mass coral bleaching
Source: Ecol Lett. 2024 Dec 31;27(12):e14454. doi: 10.1111/ele.14454 (PMC11686943; doi:10.1111/ele.14454)
Supplement: Supplementary file 6 — Table S1. [file ELE-27-0-s002.docx]

**Supplemental Table S1** Mean biomass (kg / ha) of the top 5 herbivore species in each year and regime state (lower and upper are 2 * S.E.M.). Missing values indicate that a species was not in the top 5 biomass for that regime state and year, but may have been present in lower biomass. Biomass ranked from dark green to yellow in decreasing biomass contribution within each year and regime state.

|  |  |  | Recovering | | | Shifted | | |
| --- | --- | --- | --- | --- | --- | --- | --- | --- |
| **Year** | **Species** | **Functional Group** | **Mean** | **Lower** | **Upper** | **Mean** | **Lower** | **Upper** |
| 1994 | *Chlorurus sordidus* | Excavator | 33.1 | 25.5 | 40.7 | 37.3 | 25.9 | 48.7 |
|  | *Ctenochaetus striatus* | Grazer | 21.7 | 12.2 | 31.1 | - | - | - |
|  | *Scarus niger* | Scraper | 17.1 | 11.2 | 23.1 | 13.9 | 5.7 | 22.2 |
|  | *Plectroglyphidodon lacrymatus* | Grazer | 8.5 | -3.2 | 20.3 | 16.7 | 0.1 | 33.3 |
|  | *Chlorurus strongylocephalus* | Excavator | 8.1 | 4.7 | 11.4 | - | - | - |
|  | *Hipposcarus harid* | Scraper | - | - | - | 27.2 | -4.6 | 59.0 |
|  | *Scarus prasiognathos* | Scraper | - | - | - | 14.1 | 8.1 | 20.2 |
| 2005 | *Chlorurus sordidus* | Excavator | 33.8 | 21.4 | 46.3 | 34.7 | 19.5 | 49.9 |
|  | *Scarus prasiognathos* | Scraper | 27.9 | 10.2 | 45.6 | - | - | - |
|  | *Scarus niger* | Scraper | 26.2 | 16.7 | 35.7 | 21.4 | 1.0 | 41.9 |
|  | *Scarus rubroviolaceus* | Scraper | 20.4 | 7.4 | 33.4 | - | - | - |
|  | *Hipposcarus harid* | Scraper | 13.3 | -10.8 | 37.5 | 12.8 | 0.4 | 25.2 |
|  | *Scarus ghobban* | Scraper | - | - | - | 16.0 | 6.5 | 25.5 |
|  | *Chlorurus atrilunula* | Excavator | - | - | - | 7.8 | 2.1 | 13.6 |
| 2008 | *Scarus rubroviolaceus* | Scraper | 43.4 | 13.3 | 73.5 | - | - | - |
|  | *Chlorurus sordidus* | Excavator | 29.8 | 19.6 | 39.9 | 35.8 | 18.2 | 53.3 |
|  | *Scarus niger* | Scraper | 24.9 | 13.3 | 36.5 | 23.0 | -2.9 | 48.9 |
|  | *Scarus caudofasciatus* | Scraper | 16.7 | 3.8 | 29.5 | - | - | - |
|  | *Acanthurus tennentii* | Grazer | 13.3 | 1.7 | 24.9 | - | - | - |
|  | *Scarus prasiognathos* | Scraper | - | - | - | 11.5 | -5.2 | 28.3 |
|  | *Siganus puelloides* | Grazer | - | - | - | 11.1 | -3.1 | 25.4 |
|  | *Chlorurus atrilunula* | Excavator | - | - | - | 10.2 | 1.9 | 18.6 |
| 2011 | *Scarus rubroviolaceus* | Scraper | 31.7 | 13.1 | 50.3 | - | - | - |
|  | *Chlorurus sordidus* | Excavator | 27.6 | 19.5 | 35.7 | 37.3 | 11.2 | 63.4 |
|  | *Scarus niger* | Scraper | 26.4 | 14.2 | 38.7 | 17.0 | -2.8 | 36.7 |
|  | *Scarus prasiognathos* | Scraper | 20.5 | 5.2 | 35.7 | 17.4 | 3.3 | 31.4 |
|  | *Chlorurus atrilunula* | Excavator | 12.7 | 5.6 | 19.8 | 10.3 | -1.2 | 21.8 |
|  | *Scarus ghobban* | Scraper | - | - | - | 9.2 | 0.2 | 18.2 |
| 2014 | *Chlorurus sordidus* | Excavator | 38.3 | 24.9 | 51.8 | 30.5 | 10.8 | 50.2 |
|  | *Scarus falcipinnis* | Scraper | 18.8 | -14.7 | 52.2 | - | - | - |
|  | *Scarus niger* | Scraper | 18.3 | 9.9 | 26.8 | 14.5 | 0.3 | 28.8 |
|  | *Chlorurus atrilunula* | Excavator | 10.5 | 3.6 | 17.4 | - | - | - |
|  | *Scarus rubroviolaceus* | Scraper | 9.1 | 3.1 | 15.0 | 9.0 | 2.9 | 15.0 |
|  | *Siganus sutor* | Scraper | - | - | - | 15.6 | -10.6 | 41.8 |
|  | *Chlorurus strongylocephalus* | Excavator | - | - | - | 8.7 | -2.3 | 19.8 |
| 2017 | *Chlorurus sordidus* | Excavator | 52.9 | 30.6 | 75.1 | 32.1 | 13.2 | 51.1 |
|  | *Scarus niger* | Scraper | 26.6 | 19.4 | 33.7 | 9.3 | -2.7 | 21.3 |
|  | *Scarus rubroviolaceus* | Scraper | 23.9 | 5.3 | 42.5 | 13.5 | 4.9 | 22.1 |
|  | *Scarus prasiognathos* | Scraper | 9.8 | 2.4 | 17.2 | - | - | - |
|  | *Scarus caudofasciatus* | Scraper | 8.9 | 1.1 | 16.7 | - | - | - |
|  | *Leptoscarus vaigiensis* | Browser | - | - | - | 13.0 | 5.4 | 20.6 |
|  | *Siganus sutor* | Browser | - | - | - | 11.5 | -2.3 | 25.3 |
| 2022 | *Scarus niger* | Scraper | 35.9 | 20.9 | 50.9 | 15.3 | 3.2 | 27.3 |
|  | *Chlorurus sordidus* | Excavator | 31.0 | 20.8 | 41.2 | 18.6 | 6.4 | 30.8 |
|  | *Chlorurus strongylocephalus* | Excavator | 15.8 | -2.0 | 33.6 | - | - | - |
|  | *Scarus rubroviolaceus* | Scraper | 14.3 | 3.5 | 25.0 | - | - | - |
|  | *Chlorurus atrilunula* | Excavator | 10.2 | 3.9 | 16.4 | - | - | - |
|  | *Siganus sutor* | Browser | - | - | - | 21.1 | 2.7 | 39.5 |
|  | *Scarus prasiognathos* | Scraper | - | - | - | 9.0 | 0.1 | 17.9 |
